# Load and speed effects on the cervical flexion relaxation phenomenon

**DOI:** 10.1186/1471-2474-11-46

**Published:** 2010-03-10

**Authors:** Jean-Philippe Pialasse, Danik Lafond, Vincent Cantin, Martin Descarreaux

**Affiliations:** 1 Département des sciences de l'activité physique, Université du Québec à Trois-Rivières, Trois-Rivières, Québec, Canada; 2 Département de chiropratique, Université du Québec à Trois-Rivières, Trois-Rivières, Québec, Canada; 3 Institut Franco-Européen de Chiropratique, Paris, France

## Abstract

**Background:**

The flexion relaxation phenomenon (FRP) represents a well-studied neuromuscular response that occurs in the lumbar and cervical spine. However, the cervical spine FRP has not been investigated extensively, and the speed of movement and loading effects remains to be characterized. The objectives of the present study were to evaluate the influence of load and speed on cervical FRP electromyographic (EMG) and kinematic parameters and to assess the measurement of cervical FRP kinematic and EMG parameter repeatability.

**Methods:**

Eighteen healthy adults (6 women and 12 men), aged 20 to 39 years, participated in this study. They undertook 2 sessions in which they had to perform a standardized cervical flexion/extension movement in 3 phases: complete cervical flexion; the static period in complete cervical flexion; and extension with return to the initial position. Two different rhythm conditions and 3 different loading conditions were applied to assess load and speed effects. Kinematic and EMG data were collected, and dependent variables included angles corresponding to the onset and cessation of myoelectric silence as well as the root mean square (RMS) values of EMG signals. Repeatability was examined in the first session and between the 2 sessions.

**Results:**

Statistical analyses revealed a significant load effect (P < 0.001). An augmented load led to increased FRP onset and cessation angles. No load × speed interaction effect was detected in the kinematics data. A significant load effect (P < 0.001) was observed on RMS values in all phases of movement, while a significant speed effect (P < 0.001) could be seen only during the extension phase. Load × speed interaction effect was noted in the extension phase, where higher loads and faster rhythm generated significantly greater muscle activation. Intra-session and inter-session repeatability was good for the EMG and kinematic parameters.

**Conclusions:**

The load increase evoked augmented FRP onset and cessation angles as well as heightened muscle activation. Such increments may reflect the need to enhance spinal stability under loading conditions. The kinematic and EMG parameters showed promising repeatability. Further studies are needed to assess kinematic and EMG differences between healthy subjects and patients with neck pain.

## Background

The flexion relaxation phenomenon (FRP) is commonly defined as a decrease in superficial paraspinal muscle electromyography (EMG) signals that occur just before full trunk flexion. This myoelectric silence, first observed in the lumbar region, is believed to be caused by a transfer of the extension moment from active superficial paraspinal muscles to passive viscoelastic structures of the spine, such as the ligaments, capsules and vertebral discs [1-3]. A second hypothesis suggests that, during the FRP, the trunk extension moment is transferred from the superficial paraspinal muscles to the deep lateral paraspinal muscles and quadratus lumborum muscles [4,5]. Finally, a third hypothesis proposes that reflexive mechanisms involving tension mechanoreceptors in ligaments and other viscoelastic structures trigger the FRP [6]. This hypothesis, however, was ruled out by Olson et al. (2006) implementing a task where participants completed full trunk flexion from the supine to the sitting position. These authors concluded that, although tension was generated in posterior structures, no FRP was seen in this condition, and reflexive mechanisms involving tension mechanoreceptors could not solely account for the flexion relaxation responses [7].

Recent studies have explored the relationship between lumbar spine loading and the neuromuscular neutral zone (NNZ) [8-10]. This model states that normal function and stabilization of the lumbar spine within a small movement or in the absence of extra loading only require muscular activity. Outside the NNZ, spinal stabilization is achieved with the heightened tension generated in viscoelastic structures. The FRP may very well be explained in light of the NNZ model.

Several factors, such as speed of execution and loading of the spine, have been shown to influence the FRP. Indeed, faster trunk flexion and extension as well as increased loading of the spine result in prolonged paraspinal muscle activity [11-13]. Only Sarti et al. (2001) did not find significant changes in FRP kinematic parameters after spinal loading [14]. Finally, repetition of movement can also influence the FRP. This modulation is likely the outcome of the task repetition effect on passive tissue viscoelasticity rather than muscular fatigue as the same result is obtained after passive and active repetition [15,16]. Experiencing pain is also a modulating factor as patients with lumbar pain do not demonstrate EMG silence [1,17-19]. Pain acting as a modulating factor could be explained by the fear avoidance theory [20-22] and mechanisms involving segmental reflexes and supra-segmental integration [23].

The FRP has also been described in the cervical spine [24-28]. It can be observed through a flexion/extension task of the cervical spine in a lumbo-pelvic sitting position [25,26]. Cervical FRP onset and cessation angles, estimated respectively at 74.5% and 92.5%, are not modified by trunk inclination in sitting postures [28]. Recent studies have revealed that the cervical FRP may not be seen in patients with neck pain [24] and, to our knowledge, there are no data on speed and loading effects on the cervical FRP. Moreover, very limited information is currently available regarding cervical FRP EMG and kinematic parameter repeatability [29].

Therefore, as evaluated previously in the lumbar region, kinematics and EMG parameters of the cervical FRP in healthy and neck pain subjects need to be investigated. Consequently, the objectives of this study were two-fold: firstly to evaluate the effect of load and speed on cervical FRP EMG and kinematic parameters, and secondly to assess the measurement of cervical FRP kinematic and EMG parameter repeatability. It was hypothesized that increased loading or speed will lead to greater cervical FRP onset and cessation angles.

## Methods

### Participants

Eighteen healthy adults (6 women and 12 men), aged 20 to 39 years with mean (standard deviation) age 27.2 (5.2) years, height 1.73 (0.08) m, weight 67.4 (11.3) kg, and body mass index 22.5 (2.5) kg m^-2^, participated in this study. All study subjects gave their informed, written consent according to the protocol approved by the Human Research Ethics Committee at *Université du Québec à Trois-Rivières*. Individuals with present or past neck pain, spinal trauma (including whiplash) or cervical spine surgery were excluded from the experiment.

### Experimental protocol

**Figure 1:**
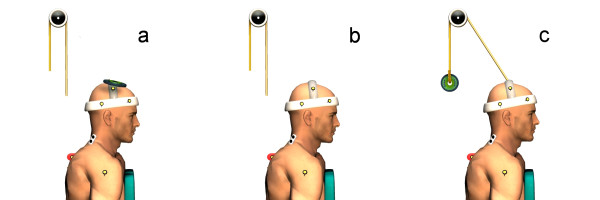
**Experimental setting of a participant in loaded (a), no load (b) and counterweighted (c) conditions**.

Participants were tested in two 60-minute experimental sessions on two separate days (24 to 48 hours). During these sessions, they were asked to perform a standardized cervical flexion/extension movement divided into 3 phases: firstly a complete cervical flexion (flexion phase), secondly a static period in complete cervical flexion (full-flexion phase) and thirdly an extension with return to the initial position (extension phase). Two different rhythms were included for this movement. The slow rhythm condition consisted of: 5-second flexion, 3-second full flexion, and 5-second extension. The fast rhythm comprised: 2-second flexion, 3-second full flexion, and 2-second extension. Different loads were applied under the following conditions: (a - loaded) 700 g, (b - no load) none, and (c - counterweighted) counterweight of -300 g, as shown in Figure 1. To ensure standardized speed of movement and to reduce intra-subject and inter-subject variability, a sound signal generated by a metronome guided participants throughout each condition. Subjects executed 3 trials of each combined load and rhythm conditions. They were allowed 2 practice trials to perform the flexion/extension task at the predefined pace and also completed a maximal cervical flexion trial that was used as a reference for flexion range of motion and EMG normalization. As a result they had to perform 21 trials: 18 FRP trials (3 × 3 × 2), two practice trials and a maximal flexion trial. A rest period was allowed between 2 rhythm series. To limit sequence effects, the test condition order of presentation was randomized for each participant.

### Instrumentation

Kinematic data were collected by a motion analysis system (Optotrak Certus, Northern Digital Inc., Waterloo, Ontario, Canada). As illustrated in Figure 1, infrared diodes were positioned on the following anatomical sites: the T2 spine process and right acromion, with 3 other diodes set up in triangle on the lateral right side of a helmet (72 g). Kinematic data were collected at a frequency of 100 Hz, and low-pass filtered by a dual-pass, fourth-order Butterworth filter with a cut-off frequency of 5 Hz. Cervical flexion angle was measured from the line pulled between 2 markers on the helmet, and a vertical line traced from the T2 marker.

Surface EMG electrodes (DE 2.1 single-differential, parallel-bar configuration, common mode rejection ratio of 92dB at 60 Hz, Delsys, Inc., Boston, MA) were applied bilaterally on the paraspinal muscles at 2 cm lateral of the C4 spinous process [25,30]. Ground electrode was applied on the left acromion. The electrodes were positioned in the direction of the muscle fibres, and skin impedance was reduced by removing the hair, by abrasion (3 M Red Dot Trace Prep, St. Paul, MN) and rubbing the skin with an alcohol-soaked compress. EMG data were collected by the Delsys Bagnoli EMG system (Bagnoli-8 channels, Delsys, Inc., Boston, MA) and sampled at 900 Hz, with a 32-bit A/D converter (PCI-6284 M SERIES DAQ, National Instruments, Austin, TX).

EMG and kinematics data were synchronized using an automatic trigger. The EMG data were filtered at a bandwidth of 10 to 450 Hz and rectified. The kinematics data were corrected for missing values with a cubic spline interpolation, and then processed with a fourth-order Butterworth filter at a frequency of 5 Hz. The data were analyzed with MatLab^® ^version 7.2 (Mathworks Inc., Natick, MA).

### Data analysis

The EMG data were filtered, and RMS values for each of the 3 phases were obtained. Normalized RMS values were computed, with an active head extension (from full-flexion) muscle burst as reference. Both non normalized and normalized angles were analyzed. Angles corresponding to EMG signal reduction during the flexion phase (FRP onset angle) and to EMG signal increment during the extension phase (FRP cessation angle) were identified by visual inspection of the squared EMG signals with complete blinding of the experimental conditions [25,28]. Theses angles were analyzed in both degrees and percentage of maximal full-flexion. The EMG signals were raised to squared values to help visual inspection. The presence or absence of FRP responses was determined visually for each trial, searching for eccentric extensor muscle activity, followed by a period of lesser activity ending with concentric extensor muscle activity. Inflexion points on the kinematics graph were identified to distinguish the different movement phases. Maximal flexion amplitude was calculated with the maximal flexion angle obtained minus participant mean neutral position (mean angle at the neutral position before each trial). Mean left and right RMS values served to assess load and speed effects during all movement phases.

### Statistical analysis

Dependent variables included FRP onset and cessation angles as well as mean RMS values. Right and left RMS and angle values were compared by student's T-test, and as there was no statistical difference, the mean values of each trial were included for all EMG analyses. Dependent variables were compared across different experimental conditions, in each phase of movement or for each angles, with 2 × 3 (speed × load) repeated-measures ANOVA, which was also performed to assess the effect of speed and loading movement on phase duration. Post-hoc analyses were undertaken with the Tukey test, and the statistical significance level was set at P < 0.05 for all analyses. Intra-class correlations (ICC) were conducted to evaluate intra- and inter-session reliability of onset and cessation angles and RMS values. Two-way random single measures (ICC(2,1)) were employed with the Spearman-Brown formula for stepped-up reliability to estimate the number of trials (k) required to obtain the expected reliability [31]. The following equation allowed estimation of the reliability coefficient of the mean (R_k_) by averaging k trials with a 1-measure reliability coefficient R:

We also estimated the number of trials (k) averaged to obtain a target coefficient of reliability (R*) with the following formula:

The number of trials (k) needed to obtain an average-measure ICC of at least .80 was calculated. Two-way random average measures (ICC(2,k)) were calculated to assess inter-session repeatability.

## Results

**Figure 2:**
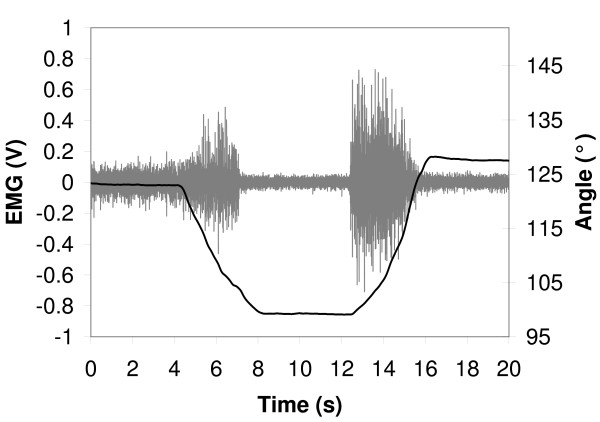
**Typical subject EMG (grey) and cervical flexion angle (black) during a slow-speed-without-load trial**.

All participants expressed a cervical FRP as defined initially and as presented in Figure 2. Although a metronome standardized movement velocity and guided study participants throughout the different movement phases, the duration of each movement phase was computed and no significant load effect was found during the flexion and extension phases. Besides, a statistically significant difference was observed between the two rhythm conditions in both flexion and extension phases. Mean movement phase durations for each condition are presented in Table 1.

**Table 1:** Mean (SD) duration of each phase in the 2 rhythm conditions

Speed	Load	Flexion phase	Full-flexion phase	Extension phase
S	a	4.9 (0.5)	3.3 (0.3)	4.8 (0.7)
	b	4.8 (0.5)	3.4 (0.3)	4.6 (0.3)
	c	4.7 (0.4)	3.5 (0.3)	4.6 (0.4)
F	a	2.1 (0.5)	3.0 (0.4)	2.2 (0.4)
	b	2.1 (0.5)	3.1 (0.4)	2.1 (0.4)
	c	2.0 (0.5)	3.1 (0.4)	2.1 (0.4)

Slow (S) and fast (F) speed conditions and loaded (a), no load (b) and counterweighted (c) load conditions are presented. Durations are reported in seconds.

### Kinematics

**Figure 3:**
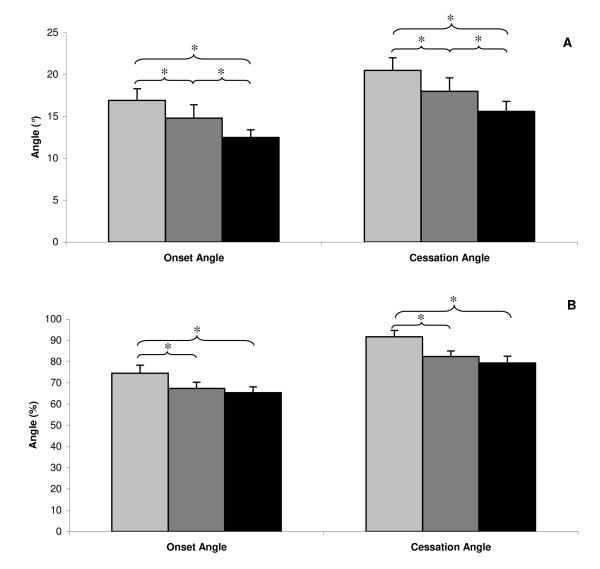
**Load effect on onset and cessation angles**. A - Illustrates mean (SE) angles expressed in degrees. Light grey represents 700 g weighted, dark grey, no weight, and black, -300 g counterweighted conditions. B - Illustrates mean (SE) angles in percentages. Light grey represents 700 g weighted, dark grey, no weight, and black, -300 g counterweighted conditions.

Repeated-measures ANOVA yielded a significant load effect on both cervical FRP cessation and onset angles (P < 0.001). Mean (Standard Error) angles for loaded, no load, and counterweighted conditions were respectively 17° ± 1.4, 15° ± 1.1 and 13° ± 0.9 with F(2,34) = 34.6, P < 0.001 for onset and 21° ± 1.5, 18° ± 1.3 and 16° ± 1.2 with F(2,34) = 45.0, P < 0.001 for cessation (Figure 3). Post-hoc analyses revealed a significant (P < 0.001) difference on both onset and cessation angles between each of the 3 load conditions. The increase in load caused a significant increment of onset and cessation angles. The decrease in load (counterweight) led to a significant decline of onset and cessation angles. Similar results were obtained in percentages: 75% ± 3.8, 67% ± 2.9 and 65% ± 2.7 with F(2,34) = 10.3, P < 0.001 for onset angle and 92% ± 3.0, 82% ± 2.6 and 79% ± 3.2 with F(2,34) = 30.0, P < 0.001 for cessation angle (Figure 3). Although post-hoc analyses revealed only a significant (P < 0.01) difference on both onset and cessation angles in percentage between loaded and the 2 other conditions.

**Figure 4:**
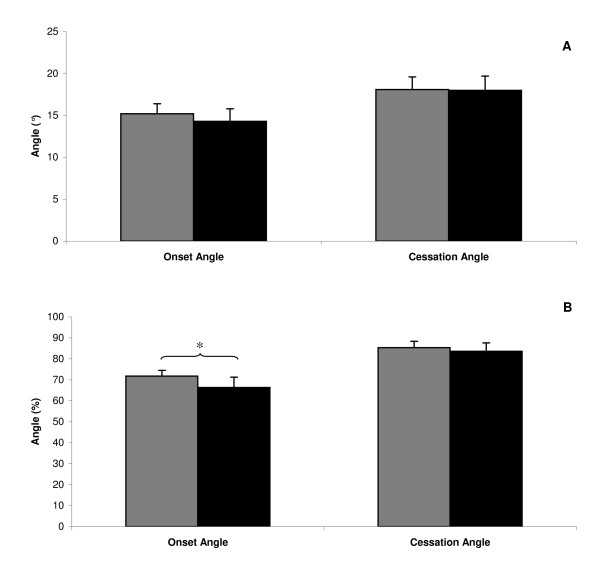
**Speed effect on onset and cessation angles**. A - Illustrates mean (SE) angles expressed in degrees. Grey represents the slow speed condition, and black, the fast speed condition. B - Illustrates mean (SE) angles expressed in percentages. Grey represents the slow speed condition, and black, the fast speed condition.

No significant speed effect was noted on both onset (15° ± 1.2 with slow speed, 14° ± 1.5 with fast speed F(1,17) = 2.7, P = 0.12) and cessation (18° ± 1.5 with slow speed, 18° ± 1.7 with fast speed F(1,17) = 0.1, P = 0.81) angles. Speed effect on FRP onset and cessation angles is presented in Figure 4. No speed × load interaction was noted on both onset (F(2,34) = 0.1, P = 0.94) and cessation angles (F(2,34) = 0.1, P = 0.90). Furthermore, analyses performed with angles expressed as a percentage of maximum flexion amplitude yielded similar results with the exception of a significant speed effect (F(1,17) = 4.8, P < 0.05) on onset angle. Mean onset angle was 72% ± 2.7 with slow movement versus 66% ± 4.9 with fast movement, and cessation angle was 85% ± 3.0 versus 84% ± 4.0, respectively (Figure 4). Increasing speed significantly decreased onset angle when it was expressed as a percentage of maximum flexion amplitude.

### EMG

Repeated-measures ANOVA disclosed a significant load effect on flexion, full-flexion and extension phase on normalized RMS values. In the flexion phase (F(2,34) = 4.1, P < 0.05), increased load raised RMS values, and decreased load (counterweight) reduced them. Post-hoc analyses discerned a significant (P < 0.05) difference only between loaded and counterweighted conditions. During the full-flexion phase (F(2,34) = 6.1, P < 0.01), increased load caused an increment of RMS values, and decreased load (counterweight) produced a slight elevation of these values. Post-hoc analyses showed significant (P < 0.05) differences between each condition, except between the no load and counterweight conditions. Finally, during the extension phase (F(2,34) = 13.9, P < 0.001), a load increase elicited an augmentation of RMS values, and a load decrease evoked a slight reduction. Post-hoc analyses demonstrated significant (P < 0.001) differences between each condition except between the no load and counterweight conditions.

**Figure 5:**
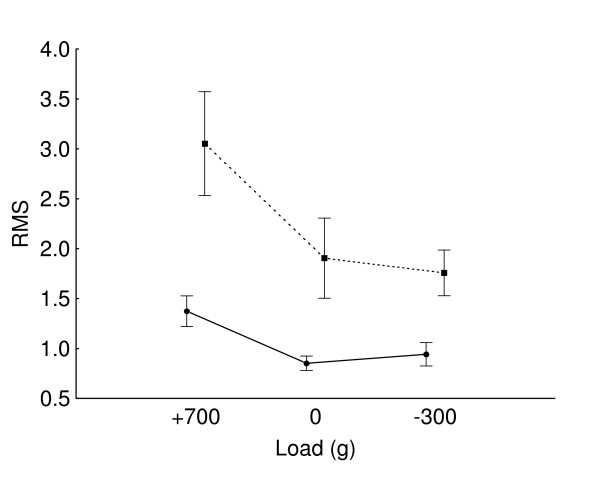
**Load × speed interaction effect on extension phase normalized EMG RMS**. Vertical bars denote standard errors. High speed is represented by the interrupted line, and slow speed, by the continuous line.

A significant (F(1,17) = 14.9, P < 0.01) speed effect was observed only in the extension phase where increasing speed augmented RMS values. Significant speed × load interaction was noted for RMS values in the extension phase (F(2,34) = 5.0, P < 0.05) (Figure 5).

### Intra-session repeatability

ICCs were computed for both RMS and normalized RMS values. RMS values systematically yielded higher ICCs, and are therefore presented in the 2 repeatability sections. Onset and cessation angles ICC(2,1) were 0.73 CI 95% [0.65-0.79] and 0.90 CI 95% [0.86-0.93] needing, respectively, 2 and 1 trials to obtain 0.80 ICC(2,k). These results were obtained with all trials pooled. A breakdown of ICC(2,1) for each of the 6 conditions is presented in Table 2.

**Table 2:** Intra-session kinematic ICC(2,1)

	Speed	Load	Mean (SD)	SEM	ICC(2,1)	CI 95% -	CI 95% +	k ICC≥80
Onset angle	S	a	17 (4.5)	2.4	0.72	0.49	0.87	2
		b	15 (3.8)	2.1	0.71	0.48	0.87	2
		c	13 (2.6)	1.7	0.64	0.38	0.83	3
	F	a	17 (5.5)	3.0	0.70	0.47	0.86	2
		b	14 (4.0)	2.1	0.73	0.51	0.88	2
		c	12 (4.2)	2.6	0.61	0.35	0.81	3
Cessation angle	S	a	21 (4.6)	1.5	0.89	0.78	0.95	1
		b	18 (4.1)	1.5	0.86	0.73	0.94	1
		c	16 (3.6)	1.56	0.81	0.64	0.92	1
	F	a	21 (5.1)	1.6	0.91	0.81	0.96	1
		b	18 (4.3)	1.2	0.92	0.84	0.97	1
		c	15 (4.2)	1.6	0.86	0.73	0.94	1

Presented with mean and standard deviations (SD), in degrees, standard error of the measurement (SEM), ICC(2,1) with 95% confidence interval, and number of trials needed to obtain an ICC(2,k) of at least .80. Slow (S) and fast (F) speed conditions and loaded (a), no load (b) and counterweighted (c) load conditions are presented.

The ICC(2,1) for RMS was 0.76 CI 95% [0.69-0.82] in the flexion phase, 0.71 CI 95% [0.63-0.78] in the full-flexion phase, and 0.86 CI 95% [0.81-0.89] in the extension phase; as a result, 2, 2 and 1 trials were respectively required to achieve 0.80 ICC(2,k) for each movement phase. In the 6 different conditions, ICC(2,1) values were between 0.53 and 0.87 for the flexion phase, 0.41 and 0.81 for the full-flexion phase, and 0.74 and 0.99 for the extension phase, needing respectively 1 to 4 trials, 1 to 6 trials, and 1 to 2 trials to obtain a 0.80 ICC(2,k).

### Inter-session repeatability

Two-way random average measures, ICC(2,k), in all conditions pooled were 0.79 CI 95% [0.70-0.86] for onset angle, and 0.85 CI 95% [0.79-0.86] for cessation angle. A breakdown of ICC for each of the 6 conditions is given in Table 3.

**Table 3:** Inter-session kinematics ICC(2,k)

	Speed	Load	Mean (SD)	SEM	ICC(2,k)	CI 95% -	CI 95% +
Onset angle	S	a	18 (4.9)	2.0	0.83	0.53	0.93
		b	15 (3.9)	2.2	0.68	0.13	0.88
		c	13 (2.5)	1.8	0.52	-0.29	0.82
	F	a	17 (5.1)	2.4	0.78	0.42	0.92
		b	14 (3.9)	2.4	0.63	0.00	0.86
		c	12 (3.5)	2.1	0.64	0.03	0.86
Cessation angle	S	a	21 (5.1)	1.7	0.88	0.68	0.96
		b	18 (3.9)	2.0	0.74	0.30	0.90
		c	16 (3.3)	1.6	0.75	0.33	0.91
	F	a	21 (5.2)	2.0	0.85	0.59	0.94
		b	18 (4.3)	2.1	0.76	0.37	0.91
		c	15 (3.9)	1.8	0.79	0.44	0.92

Presented with mean and standard deviations (SD), in degrees, standard error of the measurement (SEM), and ICC(2,k) with 95% confidence interval. Slow (S) and fast (F) speed conditions and loaded (a), no load (b) and counterweighted (c) load conditions are presented.

Two-way random average measures, ICC(2,k), for RMS were 0.84 CI 95% [0.77-0.89] in the flexion phase, 0.60 CI 95% [0.42-0.73] in the full-flexion phase, and 0.96 CI 95% [0.94-0.97] with all conditions pooled. In the 6 different non-pooled conditions, ICC(2,k) values were between 0.74 and 0.90 for the flexion phase, 0.22 and 0.93 for the full-flexion phase, and 0.94 and 0.96 for the extension phase.

## Discussion

The main objectives of this study were to assess the effect of load and speed on the cervical FRP, and to document its repeatability. The results showed that increased load significantly augmented cervical FRP onset and cessation angles as well as RMS values in all phases of the flexion/extension cycle. Increasing speed led to increment of RMS values in the extension phase and onset angle in percentages, but had no effect on angles in degrees. Moderate to excellent repeatability for the kinematics parameters was observed in all phases. Reliability of RMS values was moderate in the flexion and full-flexion phases, and excellent in the extension phase.

### Cervical FRP parameters

The effect of increasing load on the cervical FRP, by raising EMG RMS values, was similar to what was initially described in the lumbar region [11,13]. Thuresson et al. (2005) observed a similar effect on EMG signals of the paraspinal muscles at the C2 and C7 levels in neutral and 20° flexed postures, achieving a neck flexion task with head-worn equipment [32]. While evaluating paraspinal muscle activity, they also demonstrated that the flexion moment created by the load was the critical modulating factor rather than the load itself. In the current study, care was taken with regard to load distribution to increase the flexion moment with the load condition and the extension moment with the counterweight condition. The results were only significantly different between the loaded and counterweighted conditions. Additional loading might be needed to observe significant EMG differences between the no load and loaded conditions. Still, our results are in line with the passive structures hypothesis, which suggests a transfer of the extension moment from eccentric paravertebral muscle contraction to passive viscoelastic structures [1,12,14,33].

Heightened load also had an effect on FRP onset and cessation by increasing both angles. Again, the loading effect on onset angles was similar to that described in the lumbar region as the increment in load led to a significant increase in onset angle [11,12]. It is also important to note that this effect was observed during the loading phase and that the results might have been different if the subjects had been tested after loading, as reported by Youssef et al. [10] who noted possible spinal stabilization mechanisms after loading activities. The temporal modulation of EMG onset and cessation has been described as motor control adaptation to enhance spinal stability in reaction of passive structures increased laxity [34].

Increasing speed had a significant effect on RMS values in the extension phase. The absence of a significant effect on RMS values in the flexion phase could be explained by augmented viscoelastic tissue stiffness consequent to increasing speed [35]. Furthermore, the flexion moment created with head weight (and load) might be easily opposed by tension generated in passive viscoelastic tissues. Strong viscoelastic structures, such as the nuchal ligament, may generate enough tension to counteract the head gravitational load since it has been described as a significant structural restraint to cervical spine flexion [36].

Speed had no effect on onset and cessation angles. These results are different from those reported by Sarti et al. [14] who studied the speed effect on the lumbar FRP. In their experiments, lumbar FRP onset and cessation angles increased with speed. However, when onset angle were expressed as percentages of total flexion a speed effect was observed. The significant result obtained only with normalized data may result from the lowered inter-subject variability but it also may reflect the higher level of stiffness present at greater speed of movement [35].

### Repeatability of measures and clinical outcomes

To consider the cervical FRP as a measure of "clinical interest", reliability and validity values, such as sensitivity and specificity, must be determined. Previous studies have established FRP validity in the lumbar region by showing that the lumbar FRP can distinguish healthy participants from low back pain patients with a high level of specificity (75%) and sensitivity (93%) [19]. The lumbar FRP has also been used to predict motion patterns among low back pain patients [1,17,37,38]. Neblett et al. (2003) established that abnormal motion could be predicted with a high level of specificity (100%) and sensitivity (79%) [37]. Differences between healthy participants and patients with neck pain have also been reported in the cervical region [24].

To date, only Murphy et al. (2007) reported the reliability of the cervical FRP. The reliability of the EMG root-mean-square (RMS) ratio of the flexion or extension phase (whichever was higher) divided by the full-flexion phase over 4 weeks was 0.96 95% CI [0.80-0.99] for the right extensors and 0.95 95% CI [0.80-0.99] for the left extensors [29].

In the current study, moderate to strong reliability of onset and cessation cervical FRP and cervical paraspinal muscle EMG RMS values were observed whereas in the lumbar region, reliability of RMS values ranged from good to excellent [19].

The fact that only moderate reliability was obtained for EMG RMS values in the full-flexion phase may reflect the nature and complexity of EMG signals. Nevertheless, kinematic values derived from EMG onset and cessation of the cervical FRP showed moderate to strong reliability, despite various difficulties related to EMG trial-to-trial reproducibility.

Future work should focus on the effect of muscle fatigue, age, and task repetition on cervical FRP parameters. Differences in kinematic parameters between healthy participants and patients with chronic and acute neck pain need to be thoroughly documented. Finally, treatment effects could be assessed by changes in flexion relaxation responses after different types of treatment.

### Limitations

All participants (using the visual inspection method) subjectively demonstrated a cervical FRP. However, Burnett et al. (2009) established that different criteria, to define the presence of a FRP, may lead to different conclusions regarding the presence or absence of the cervical FRP [25]. The results of the present study may have been different if another FRP criterion would have been applied. The use of an algorithm such as the integrated profile, should be considered to determine onset and cessation of silence and eccentric and concentric EMG activity [39]. Nevertheless, a standardized and blinded visual inspection method yielded strong load and speed effects with good levels of repeatability.

The most frequently used value for EMG normalization is the maximum voluntary isometric contraction (MIVC). Because the value of cervical FRP as a clinical evaluation tool will eventually involve patients with neck pain, for which MIVC may increase pain, an alternative normalization procedure was chosen (dynamic contraction) [40]. The chosen normalization procedure may, however, have affected the repeatability data.

To establish a clinically-relevant cervical FRP criterion and before any generalization to clinical population can be made, future experiments on the cervical FRP should be conducted to assess EMG and kinematic differences between healthy participants and patients with neck pain, as it was done earlier in the lumbar region [19].

## Conclusion

Although the cervical FRP seems to share similarities with what has been described in the lumbar region, it may be modulated by different factors. The results of the current work show that cervical FRP EMG and kinematic parameters can be modulated by loading the cervical spine. Speed of movement, however, does not culminate in systematic changes in cervical FRP EMG and kinematic parameters, whereas both EMG and kinematic parameters reveal promising repeatability. Future studies should investigate, as a priority, RMS values and kinematic parameter differences between healthy participants and patients with neck pain.

## Competing interests

The authors declare that they have no competing interests.

## Authors' contributions

JPP and MD participated in study design, experimentation, data analysis and manuscript writing. DL and VC helped in the study design, data analysis and manuscript drafting. All authors read and approved the final manuscript.

## Pre-publication history

The pre-publication history for this paper can be accessed here:

http://www.biomedcentral.com/1471-2474/11/46/prepub
